# Risk of rehospitalization due to *Clostridioides difficile* infection among hospitalized patients with *Clostridioides difficile*: a cohort study

**DOI:** 10.1017/ice.2024.155

**Published:** 2024-11

**Authors:** Emily N. Drwiega, Stuart Johnson, Larry H. Danziger, Andrew M. Skinner

**Affiliations:** 1 College of Pharmacy, University of Illinois Chicago, Chicago, IL, USA; 2 Edward Hines Jr., VA Hospital Research Service, Hines, IL, USA; 3 Stritch School of Medicine, Loyola University, Maywood, IL, USA; 4 Research Section and Infectious Diseases Section, VA Salt Lake City Health Care System, Salt Lake City, UT, USA; 5 School of Medicine, University of Utah, Salt Lake City, UT, USA

## Abstract

**Background:**

Reducing rehospitalization has been a primary focus of hospitals and payors. Recurrence of *Clostridioides difficile* infection (CDI) is common and often results in rehospitalization. Factors that influence rehospitalization for CDI are not well understood.

**Objective:**

To determine the risk factors that influence rehospitalization caused by CDI.

**Design:**

A retrospective cohort study from January 1, 2018, to December 31, 2018, of patients aged ≥18 who tested positive for *C. difficile* while hospitalized.

**Setting:**

Academic hospital.

**Methods:**

The risk of rehospitalization was assessed across exposures during and after the index hospitalization using a Cox proportional hazards model. The primary outcome of this study was 60-day CDI-related rehospitalization.

**Results:**

There were 559 hospitalized patients with a positive CD test during the study period, and 408 patients were included for analysis. All-cause rehospitalization was 46.1% within 60 days of the index hospital discharge. Within 60 days of discharge, 68 patients developed CDI, of which 72.5% (49 of 68) were rehospitalized specifically for the management of CDI. The risk of rehospitalization in patients with CDI was higher among patients who were exposed to systemic antibiotics ([adjusted hazard ratio] aHR: 2.78; 95% CI, 1.36–5.64) and lower among patients who had post-discharge follow-up addressing *C. difficile* (aHR: 0.53; 95% CI, 0.28–0.98).

**Conclusions:**

Exposure to systemic antibiotics increased the risk of rehospitalization due to CDI, while post-discharge follow-up decreased the risk of rehospitalization due to CDI. Comprehensive transitions of care for hospitalized patients with *C. difficile* may reduce the risk of CDI-related rehospitalization.

## Introduction

*Clostridioides difficile* is a spore-forming, gram-positive anaerobic bacteria that causes infections ranging from colonization to fulminant colitis.^1,2^ *C. difficile* infection (CDI) is the most common cause of healthcare-associated infectious diarrhea and causes significant morbidity and mortality resulting in substantial cost to the healthcare system.^3,4^ Despite resolution after treatment, CDI recurrence is common. Current estimates are that approximately 10%–30% of patients treated for CDI will have at least 1 recurrence.^5^ Risk for recurrence varies depending on initial treatment selection, administration of other systemic antibiotics, reduced immune response, advancing age, or severe underlying disease.^6^ Patients with recurrence may require rehospitalization due to the severity of the disease.^7^ Rehospitalizations are a particular focus of the Centers for Medicare and Medicaid Services (CMS), and improvements in care coordination at discharge are important to prevent recurrence and rehospitalization.^8^

Multiple studies have demonstrated the benefit of early patient follow-up after hospitalization in reducing rehospitalization, especially but not exclusively, with regard to heart failure and chronic obstructive pulmonary disease.^9^ The benefit of follow-up appointments for CDI has not been previously described. Specialty providers, such as infectious diseases (ID) or gastroenterology (GI), are uniquely trained to manage potential complex gastrointestinal infections, such as CDI. Consultations from these providers may occur during hospitalization to provide recommendations regarding initial or discharge management. In conjunction or alternatively, patients may see specialty providers following discharge from the hospital for follow-up and ongoing management of CDI. There is a growing body of evidence that while inpatient evaluation by specialists is critical for patient care for numerous diseases, rehospitalization reduction is strongly influenced by close post-discharge follow-up.^9–13^

The purpose of our study was to evaluate critical influences on rehospitalization related to CDI.

## Methods

### Study design and population

This study was a single-center, retrospective cohort study of patients who tested positive for *C. difficile* and who were admitted at a 547-bed tertiary academic medical center located outside of Chicago, Illinois, and were subsequently discharged from the hospital from January 1, 2018, through December 31, 2018. Hospital protocol allowed for *C. difficile* testing of any patient within the first 72 hours of hospitalization with a stool that nursing staff deemed as “loose” without physician review. After 72 hours, testing required an infection control review. The study was reviewed by the Loyola Institution Review Board and deemed exempt (IRB no. 213449).

### Study definitions and outcomes

A *C. difficile* event (CD event) during the index hospitalization was defined as a person having a positive *C. difficile* polymerase chain reaction (PCR) test (GI panel PCR, FilmArray, Biofire, or *C. difficile* PCR, Xpert CD assay, Cepheid). For analysis, these events were divided into primary CD event and recurrent CD event. A primary CD event was defined as a positive *C. difficile* test with no positive *C. difficile* test within 8 weeks of the index hospitalization. A recurrent CD event was defined as a positive *C. difficile* test, and the patient had a positive *C. difficile* test within 2–8 weeks prior to the index event.

The primary outcome was rehospitalization due to a CDI within 60 days of the index hospitalization discharge. CDI was defined as a positive *C. difficile* PCR within 2–8 weeks of the positive test during the index event with documented evidence of diarrhea and a clinical scenario consistent with a CDI within the electronic medical record (EMR). Patients diagnosed as having *C. difficile* are at risk for developing a recurrent case up to 8 weeks after completing therapy; thus, rehospitalization within 60 days was selected for the primary endpoint rather than within 30 days because the CMS 30-day hospital readmission metric may fail to capture all patients at risk for developing a subsequent CDI after the index hospitalization.^8,14^ The secondary outcomes were rehospitalization due to a CDI within 30 days of discharge and all-cause rehospitalization at 60 days. Patients with planned hospitalizations were excluded from all-cause rehospitalization analysis (ie, chemotherapy, elective surgery [n = 6]). CDI-related rehospitalization was defined as a patient requiring rehospitalization in which CDI was listed as a primary cause of admission in the rehospitalization discharge note.

### Data collection

The EMR was manually chart reviewed for all patients who tested positive for *C. difficile* to extract data pertaining to demographics, white blood cell (WBC) count (cells/mL), creatinine (mg/dL), temperature (Celsius), Charlson Comorbidity Index (CCI)^15^, use of proton pump inhibitors (PPI) or histamine receptor type 2 antagonist (H2RA), immunosuppressed status, admitting physician service, ID or GI inpatient consultation, post-discharge clinical follow-up, systemic antibiotic exposures up to 60 days after hospital discharge, and CDI treatment.

### Variable definitions

CDI treatment was defined as the receipt of antibiotics directed toward *C. difficile* for >48 hours. In patients that received CDI treatment, the variable was subdivided into treatment with a standard 10-day oral vancomycin regimen, an oral vancomycin taper regimen, a 10–14-day metronidazole regimen, and “other” regimens. Other regimens included fidaxomicin treatment (n = 1) and other nonstandard treatment regimens as defined by the IDSA/SHEA 2021 guidelines.^16^ Immunosuppressed was defined as a patient having a current diagnosis of solid organ malignancy, uncontrolled human immunodeficiency virus (HIV) (CD4 <200 and not currently on HIV therapy), autoimmune disease, history of organ transplantation, lymphoma, leukemia, multiple myeloma, or receipt of medications with known immunocompromising effects such as long-term steroids (eg, 20 mg of prednisone [or equivalent] for ≥20 days) for any reason not listed above. Post-discharge clinical follow-up addressing *C. difficile* was defined as any clinical follow-up within 60 days of discharge (ie, primary care provider or specialist) after the index hospitalization with EMR documentation by the provider addressing *C. difficile.* Exposure to systemic antibiotics was defined as antibiotic use that was not for CDI. Post-hospitalization data was collected up to 60 days after discharge from the hospital or until the patient was hospitalized for any cause.

### Patient exclusions

Individuals were excluded from analysis if they died or entered hospice during hospitalization, were lost to follow-up immediately upon discharge (defined as no record of any further care in the EMR up to 90 days after hospital discharge), or were placed on long-term *C. difficile* suppression beyond the study period, the CDI treatment regimen prescribed during the index event was completed less than 14 days prior to the end of the study period (60 days from patient discharge), the patient died prior to completing CDI therapy, or CDI therapy was completed >30 days prior to discharge from the hospital during the index event. Exclusion criteria were selected to reduce potential confounding for patients in which CDI could not be reasonably assessed. To assess for selection bias, baseline demographics of excluded patients were assessed separately (data not shown).

### Statistical analysis

All statistical analyses were conducted using R v4.3.1. Significance was defined as a p-value of ≤ 0.05, and all tests were two-tailed. Fisher exact test and χ^2^ test were used to assess for differences in proportions across categorical variables for individuals who developed a CDI within 60 days of the index hospital discharge and individuals who did not develop a CDI. The normality of continuous variables was determined by the Kolmogorov–Smirnov method. Wilcoxon signed-rank test was used to compare nonparametric variables, and the Student *t* test was used to compare parametric variables. Nonparametric variables were reported as median with interquartile range (IQR), and parametric variables were reported as mean with 95% CI.

Time-to-event methods were utilized to assess the effect of key variables on 60-day CDI-related rehospitalization for patients who developed CDI after the index hospitalization discharge (n = 68). Kaplan–Meier method was used to determine the unadjusted relationship between both systemic antibiotic exposure within 60 days of hospital discharge and time to rehospitalization as well as post-discharge clinical follow-up and time to rehospitalization. Individuals were censored on the day that they died, entered hospice, were lost to follow-up, or were rehospitalized for any cause. Log-rank test was used to assess the difference between the survival curves.

Cox proportional hazard regression methods were utilized to estimate the crude and adjusted hazard ratios (cHR and aHR) reported with 95% CI for variables that were determined to reasonably affect rehospitalization related to CDI in patients who developed a CDI. Time-zero for the Cox proportional hazard model was defined as the day of index hospitalization discharge.

The variables that were deemed to reasonably affect rehospitalization related to CDI included during the index hospitalization were (1) age ≥65 years old, (2) a stratified CCI (CCI: ≤1, 2–3, and ≥4), (3) serum WBC (cells/mL), (4) serum creatinine (mg/dL), (5) immunosuppressed status, (6) index case classified as recurrent CD event, (7) inpatient admitting service (medical service), (8) treatment of *C. difficile*, (9) ID consultation while inpatient, (10) GI consultation while inpatient, (11) in-person post-discharge clinical follow-up appointment for *C. difficile* after index event, and (12) receipt of systemic antibiotics prior to rehospitalization or up to the end of 60-day study period in patients not rehospitalized.

The multivariable model included all variables that were significant in univariate analysis. Additionally, we included the stratified CCI variable due to a known risk of all-cause rehospitalization associated with a higher CCI and because multiple components of the CCI are known risk factors for CDI (ie, age, immunosuppression, renal disease).^17,18^ To reduce the potential for multicollinearity with CCI, the variables age ≥65, serum creatinine, and immunosuppressed status were excluded from the multivariable model. The remaining variables were added in a stepwise fashion and compared by –2 log-likelihood comparison. The model that provided the best fit was chosen as the final model. Model assumptions were assessed via Schoenfeld residuals and log-log plots.

## Results

**Figure 1.**
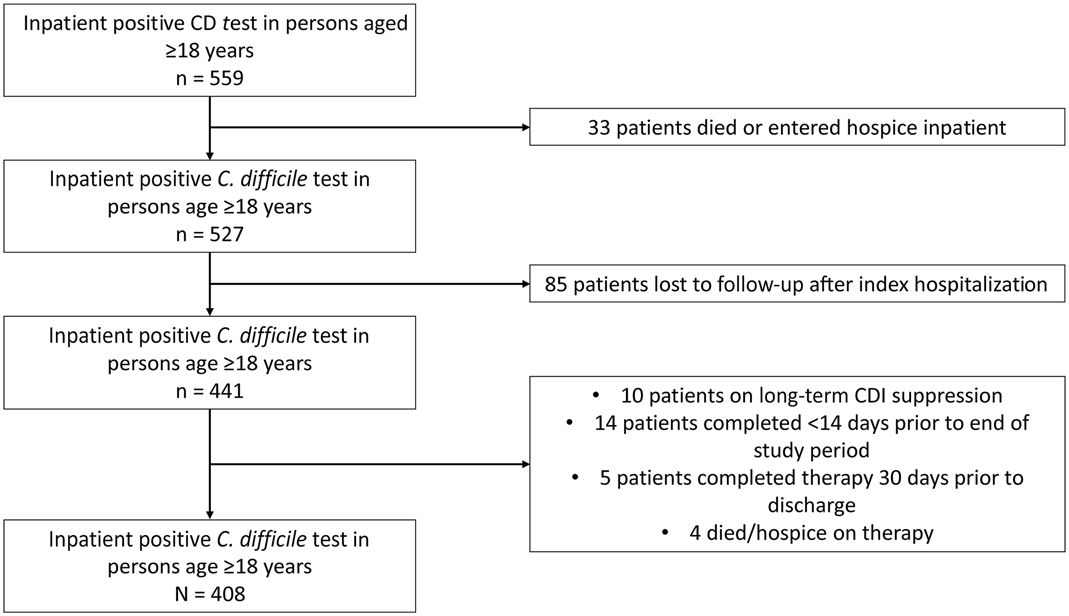
Participant selection flowsheet. CD, *C. difficile*; CDI, *C. difficile* infection.

From January 1, 2018, to December 31, 2018, we identified 559 individual cases of a positive *C. difficile* test during that hospitalization (Figure 1). Among these 559 cases, 33 were excluded as the individuals either died or were enrolled in hospice prior to discharge. Another 85 cases were excluded as the individuals were lost to follow-up immediately on hospital discharge. Another 33 were excluded as they were placed on long-term CDI suppression with vancomycin, a treatment course beyond the study period, died prior to completing CDI therapy or had completed CDI therapy >30 days prior to hospital discharge. After exclusion, there were 408 index cases available for review (Figure 1).

### Demographics

Of the 408 cases included for analysis, 51.2% (209 of 408) were female, and the median age was 63 (IQR: 51–72). Prior to the index event, 16.9% (69 of 408) of individuals with a case had been diagnosed as having CDI within the past 6 months, and 45.8% (187 of 408) were classified as immunosuppressed (Table 1). Primary CD events accounted for 85.8% (350 of 408) of cases, and 14.2% (58 of 408) of the index cases were classified as recurrent CD events (Table 1).

**Table 1.** Demographics

	All included participants (n = 408) No. (%)	No CDI after hospital discharge (n = 340) No. (%)	CDI within 60 days of discharge (n = 68) No. (%)	*P* value
Demographics				
Female	209 (51.2%)	175 (51.5%)	34 (50.0%)	0.82
Median age, years (IQR)	63 (51–72)	63 (51–72)	63 (51–71)	0.93
Age ≥ 65	193 (47.3%)	160 (47.1%)	33 (48.5%)	0.57
Median Charleston Comorbidity Index (IQR)	2 (1–4)	2 (1–4)	2 (1–4)	0.95
Cases with CDI in the past 6 mo	69 (16.9%)	54 (15.9%)	15 (22.1%)	0.21
Immunosuppressed	187 (45.8%)	151 (44.4%)	36 (52.9%)	0.20
Inflammatory bowel disease	19 (4.7%)	16 (4.7%)	3 (4.4%)	0.97
Exposed to systemic antibiotics prior to hospitalization	246 (60.1%)	203 (59.7%)	43 (63.2%)	0.59
PPI or H2RA use prior to hospitalization	118 (29.0%)	98 (28.8%)	20 (29.4%)	0.96
Diarrhea reported	257 (63.0%)	213 (62.6%)	44 (64.7%)	0.81
Mean WBC (95% CI)	9.33 (8.69–9.97)	9.41 (8.70–10.12)	8.95 (7.47–10.43)	0.61
Mean creatinine (95% CI)	1.79 (1.59–2.01)	1.83 (1.58–2.08)	1.64 (1.33–1.96)	0.52
Mean temperature, C (95% CI)	36.89 (36.84–36.95)	36.88 (36.83–36.94)	36.96 (36.80–37.12)	0.31
Severe CDI† (%)	169 (41.4%)	141 (41.5%)	28 (41.2%)	0.93
Treated for *C. difficile*	335 (82.1%)	282 (82.9%)	53 (77.9%)	0.42
Treated with metronidazole	17 (5.1%)	14 (5.0%)	3 (5.7%)	0.90
Treated with standard vancomycin regimen	292 (88.4%)	245 (86.9%)	47 (88.7%)	<0.01
Treated with vancomycin taper regimen	21 (6.3%)	18 (6.4%)	3 (5.7%)	0.91
Treated with other regimens	5 (0.3%)	5 (1.8%)	0 (0%)	--
Recurrent *C. difficile* case (%)	58 (14.2%)	42 (12.6%)	15 (22.1%)	0.07

No, number; %, percent; IQR, interquartile range; CDI, *C. difficile* infection; PPI, proton pump inhibitor; H2A, Histamine Type-2 Receptor Antagonist; WBC, white blood cell; †severe CDI defined as WBC >15,000 cells/mL or creatinine ≥1.5 mg/dl.

### All-cause rehospitalization

**Figure 2.**
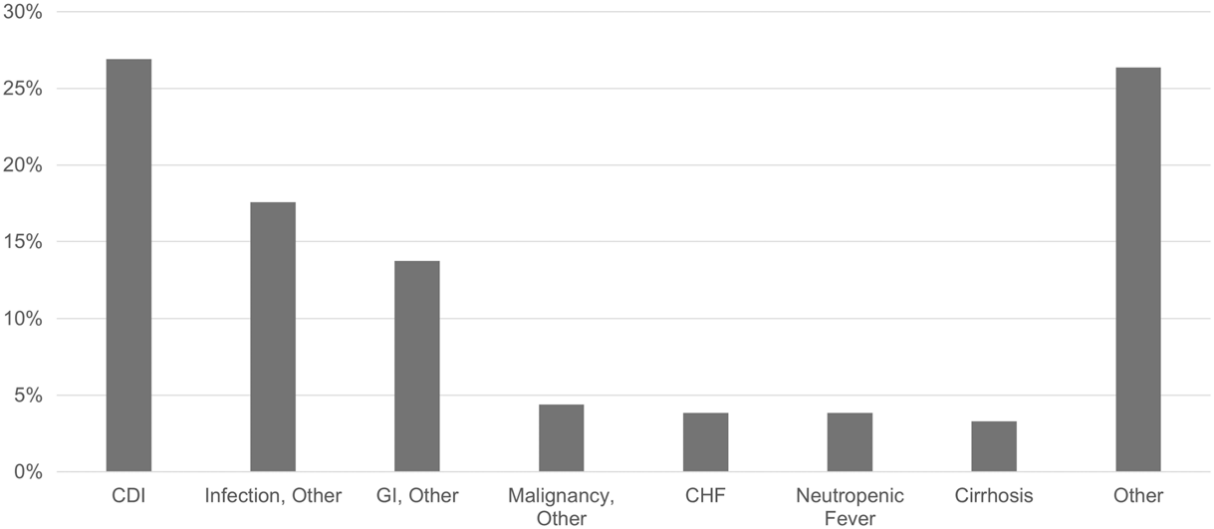
Proportion for all-cause reasons for rehospitalization. CDI, rehospitalization caused by *C. difficile* infection. Infection, Other, rehospitalization caused by non-CDI infections. GI, Other, rehospitalization caused by gastrointestinal issues, not related to CDI (ie, peptic ulcer disease, melena). Malignancy, Other, rehospitalization caused by malignancy complications, excluding planned rehospitalizations. CHF, rehospitalization caused by congestive heart failure. Neutropenic Fever, rehospitalization caused by neutropenic fever. Cirrhosis, rehospitalization caused by decompensated cirrhosis. Other, rehospitalization caused by nonspecific causes such as abnormal labs (ie, anemia), dyspnea, chest pain, fatigue, falls, or nonspecific complaints.

Among all cases, 46.1% (188 of 408) of individuals were rehospitalized for any reason within 60 days. The median time to rehospitalization for all patients was 23 days (IQR: 12–36). Among the study population, there were multiple reasons for rehospitalization. After excluding planned rehospitalization within 60 days of discharge (n = 6), the most common reason for rehospitalization at 60 days was CDI (49 of 182 [26.9%]) (Figure 2).

### Patients with CDI after discharge

For the entire study population, 68 of 408 (16.7%) had a CDI within 60 days of hospital discharge, and 49 of 68 (72.1%), or 12.2% (49 of 408), were rehospitalized within 60 days for the management of CDI. Among the 68 patients who developed CDI post-hospitalization, 64.7% (44 of 68) developed CDI within 30 days of discharge, and 72.3% (32 of 44) were rehospitalized for the management of CDI. Of 24 patients who developed a CDI between days 31 and 60, 70.8% (17 of 24) were rehospitalized for the management of CDI within 60 days of discharge.

### Risk of CDI-related rehospitalization for patients with CDI after discharge

**Figure 3.**
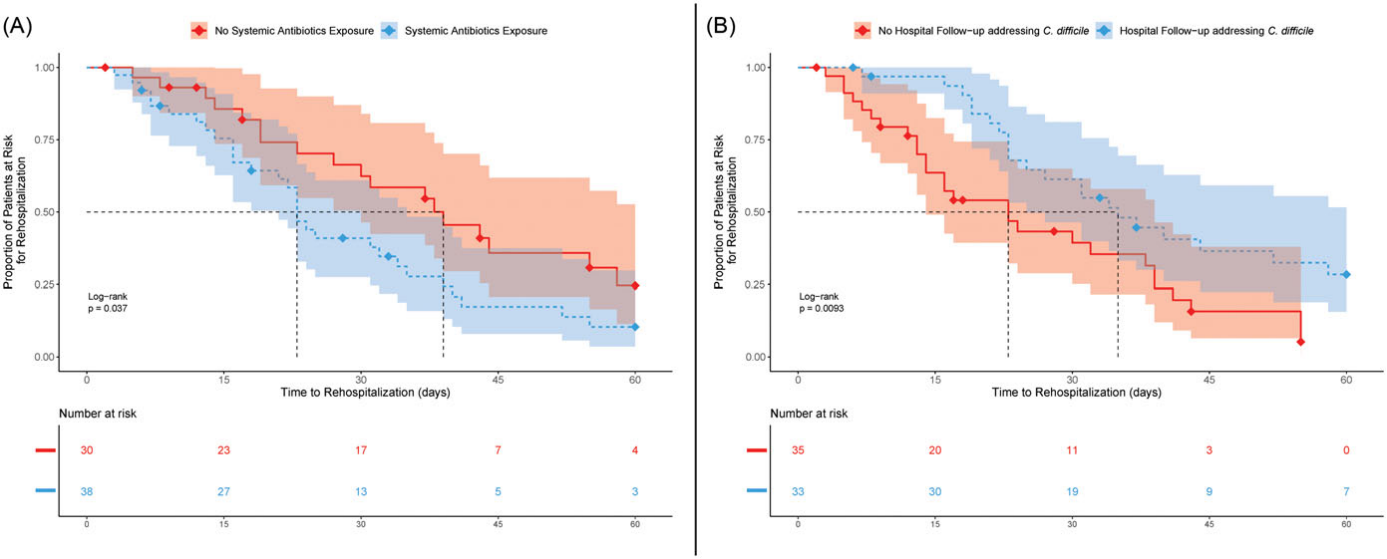
Kaplan–Meier survival curve for time to CDI-related rehospitalization in cases who developed a CDI post-discharge. (A) Systemic Antibiotics within 60 days of index discharge; (B) Hospital follow-up addressing *C. difficile*. The red line represents the lack of (A) systemic antibiotics within 60 days of index discharge and (B) hospital follow-up addressing *C. difficile*. The blue line represents the presence of (A) systemic antibiotics within 60 days of index discharge and (B) hospital follow-up addressing *C. difficile*. Diamonds represent time point in which the patient is censored. The dashed line represents the median rehospitalization time (50^th^ percentile).

The Kaplan–Meier results reveal the CDI-related rehospitalization rate was lower in individuals who had post-discharge follow-up addressing the previous positive *C. difficile* test and was higher among individuals who were exposed to systemic antibiotics (Figure 3). Univariate Cox proportional hazard results revealed multiple factors that could have affected the rate of rehospitalization among individuals who developed a CDI (n = 68). There was an increased rate of rehospitalization among persons who had either been admitted to a medical service (cHR: 3.35; 95% CI, 1.20–9.35), who were treated with metronidazole (cHR: 4.65; 95% CI, 1.37–15.80), or who were exposed to systemic antibiotics up to 60 days from index hospitalization discharge (cHR: 1.92; 95% CI, 1.06–3.44). Inversely, the rate of CDI-associated rehospitalization decreased in individuals who received a post-discharge hospital follow-up addressing CDI management in the clinic (cHR: 0.49; 95% CI, 0.28–0.88) (Table 2).

**Table 2.** Hazard ratios for CDI-related rehospitalization of patients with *C. difficile* infections after discharge

	Recurrent CDI within 60 days (n = 68) No. (%)	Crude hazard ratio (95% CI)	Adjusted hazard ratio (95% CI)
Age ≥ 65†	33 (48.5%)	1.58 (0.89–2.79)	–
Charlson Comorbidity Index			
≤1	18 (26.5%)	0.95 (0.48–1.87)	1.27 (0.57–2.83)
2–3	28 (41.2%)	REF	REF
≥4	22 (32.4%)	0.74 (0.38–1.45)	0.88 (0.42–1.83)
Mean WBC (cells/mL) (95% CI)†	8.95 (7.47–10.43)	1.02 (0.97–1.07)	–
Mean creatinine (mg/dL)†	1.64 (1.33–1.96)	1.24 (0.69–2.22)	–
Immunosuppressed†	36 (52.9%)	0.80 (0.46–1.40)	–
Epidemiologic classification†			
Primary CD event	42 (61.8%)	REF	–
Recurrent CD event	7 (10.3%)	1.42 (0.76–2.63)	–
Admitted to medical service	57 (83.8%)	3.35 (1.20–9.35)	3.62 (1.09–9.14)
CDI treatment			
Metronidazole	3 (4.4%)	4.65 (1.37–15.80)	3.86 (0.95–15.70)
Standard vancomycin regimen	47 (69.1%)	REF	REF
Vancomycin taper	3 (4.4%)	1.37 (0.42–4.52)	0.52 (0.15–1.81)
No treatment	15 (22.1%)	1.29 (0.66–2.53)	1.28 (0.64–2.58)
GI inpatient consultation†	13 (19.1%)	1.22 (0.60–2.46)	–
ID inpatient consultation†	9 (13.2%)	1.14 (0.51–2.54)	–
Hospital follow-up	33 (48.5%)	0.49 (0.28–0.88)	0.53 (0.28–0.98)
Exposure to systemic antibiotics	38 (55.9%)	1.92 (1.06–3.44)	2.78 (1.36–5.64)

No, number; %, percent; CI, confidence interval; ID, infectious diseases; GI, gastrointestinal; CDI, *C. difficile* infection; WBC, white blood cell count.

Antimicrobial dosing: metronidazole: 500 mg 3 times daily for 10–14 days; standard vancomycin regimen: 125 mg 4 times daily for 10–14 days; vancomycin Taper: 125 mg 4 times day for 10–14 days followed by vancomycin taper/pulse regimen that varied in duration by provider.

† Indication that variable was not included in final model; REF, reference group used for comparison under variable header.

After adjustment, rehospitalization rates for CDI remained increased for patients admitted to a medical service (aHR: 3.15; 95% CI, 1.09–9.14) and for patients who were exposed to systemic antibiotics (aHR: 2.78; 95% CI, 1.36–5.64). As with the univariate analysis, patients who had a post-discharge clinical follow-up in which *C. difficile* was addressed had a decreased rate of CDI rehospitalization (aHR: 0.53; 95% CI, 0.28–0.98) (Table 2).

## Discussion

Rehospitalization is an important healthcare concern and should be a priority of hospital leadership.^8^ Prior studies have evaluated the impact of post-discharge follow-up on reducing rehospitalizations following disease states other than CDI. However, there is a paucity of data evaluating the impact of post-discharge follow-up after CDI on rehospitalizations.^9^ Approximately 16% of individuals included in our study developed a CDI after hospital discharge, which is consistent with estimates previously published in the literature.^5^ The most common cause for rehospitalization was CDI, and most of the patients who developed a CDI following discharge were rehospitalized. Together, these data highlight CDI as an area for focus to reduce overall rehospitalizations.

Although only 12% of all index CD events were rehospitalized for CDI following discharge, we believe targeting post-discharge follow-up is one potential strategy to reduce CDI-related rehospitalizations. Current estimates indicate that the average rehospitalization cost is $15,200 per patient.^19^ Within our study, 118 patients had an unplanned rehospitalization within 30 days, and 182 patients had an unplanned rehospitalization within 60 days, which would result in approximately $1.8 million and $2.7 million in rehospitalization cost within 30 and 60 days of discharge, respectively. Thirty-day rehospitalization is a particular priority of CMS and, as such, is a focus of many hospitals’ leadership.^8^ Given the age and co-morbid medical conditions found in persons typically diagnosed as having CDI, they are a population at high risk for hospitalization at baseline.^18^ However, CDI can significantly affect multiple systems. Given the significant volume depletion and extracellular fluid contraction often associated with CDI diarrhea, an associated impact could be found on persons with co-morbidities such as heart failure or cirrhosis.^20,21^ These persons are often sensitive to volume shifts and would likely require close follow-up for these non-CDI-related issues. Additionally, hospitalization increases the risk of non-CDI-related infections. Although many hospitals have implemented infection control practices to minimize these risks, hospitalization places persons at risk for subsequent infections including infection due to multidrug-resistant organisms, sepsis, urinary tract infections, and pneumonia.^22,23^ Moreover, these subsequent infections continue to increase the risk of subsequent development of a CDI likely because of additional systemic antibiotic exposures.^6^ Within our study, we found that the 2^nd^ most common cause of rehospitalization was another infectious etiology. Our study was unable to capture if these cases went on to develop a further CDI, but given that our data reveal an increased rate of rehospitalization related to CDI with systemic antibiotics, these individuals were at increased risk. Further study is required to determine which systemic antibiotics increase the risk of rehospitalization.

When accounting for only CDI-related rehospitalization at 30 and 60 days post-discharge, this would equate to $486,400 and $729,600 per current rehospitalization cost estimates.^19^ Within our study, we found a hazard reduction of 47% for rehospitalization for persons who had post-discharge follow-up for CDI at 60 days. This would indicate a significant amount of savings for a hospital.

These data further show the need for a reduction in rehospitalizations related to CDI in patients initially hospitalized with a CD event. Given the increased rate of rehospitalization due to recurrence in those who received systemic antibiotics within 60 days after discharge, ensuring coordination of multispecialty care and communication may prove beneficial. Comprehensive transitions of care should be further studied, particularly in those individuals discharged from the hospital following a CD event. We believe that the key components that warrant the most attention in future studies are the benefit of discharge planning in patients with positive *C. difficile* testing, including a timely post-hospitalization clinic appointment, providing *C. difficile* medication instructions (if indicated), symptoms of *C. difficile* to monitor for, and a point of contact if they have questions. Additionally, studying the impact of reviewing antibiotics prior to discharge in this population and ensuring dedicated follow-up to confirm continued resolution would provide context into possible interventions.

Our study is not without limitations. As a retrospective study, there is the possibility of unmeasured confounding. However, a robust chart review and the interconnection of multiple EMRs from multiple hospitals allowed us to collect additional data to reduce potential confounding. Individuals were included with a positive PCR test, regardless of toxin testing, thus potentially including individuals who were colonized. However, recent data have revealed that persons who are colonized may also be at increased risk for developing a true CDI when exposed to systemic antibiotics.^24,25^ Additionally, strain typing data were not available; thus, we cannot account for the potential impact of outbreak strains with increased risk for CDI recurrence. At the time these data were collected, vancomycin was the preferred treatment option. As fidaxomicin is now the recommended treatment for persons with primary CDI and recurrent CDI, this treatment may affect recurrence rates and thus affect the number of persons rehospitalized for CDI.^16^ Lastly, our determination of hospital follow-up is reliant on narrative documentation and may not fully capture conversations between patients and providers.

## Conclusions

There are numerous facets that could affect rehospitalization related to CDI. These data indicate that dedicated hospital follow-up and antimicrobial stewardship could reduce CDI-associated rehospitalizations. Further study is required to assess these critical steps within patient transitions of care addressing *C. difficile* that is detected during hospitalization and how to best prevent rehospitalization.
